# The *OTX2* Gene Induces Tumor Growth and Triggers Leptomeningeal Metastasis by Regulating the mTORC2 Signaling Pathway in Group 3 Medulloblastomas

**DOI:** 10.3390/ijms25084416

**Published:** 2024-04-17

**Authors:** Elisabet Ampudia-Mesias, Charles S. Cameron, Eunjae Yoo, Marcus Kelly, Sarah M. Anderson, Riley Manning, Juan E. Abrahante Lloréns, Christopher L. Moertel, Hyungshin Yim, David J. Odde, Nurten Saydam, Okay Saydam

**Affiliations:** 1 Division of Hematology and Oncology, Department of Pediatrics, Medical School, University of Minnesota, Minneapolis, MN 55454, USA; ampud001@umn.edu (E.A.-M.); scameron242@gmail.com (C.S.C.); or yej980324@hanyang.ac.kr (E.Y.); moert001@umn.edu (C.L.M.); 2 Department of Pharmacy, Institute of Pharmaceutical Science and Technology, College of Pharmacy, Hanyang University, Ansan 15588, Gyeonggi-do, Republic of Korea; hsyim@hanyang.ac.kr; 3 Department of Biomedical Engineering, University of Minnesota, Minneapolis, MN 55455, USA; kell1436@umn.edu (M.K.); and05936@umn.edu (S.M.A.); manni386@umn.edu (R.M.); oddex002@umn.edu (D.J.O.); 4 University of Minnesota Informatics Institute, University of Minnesota, Minneapolis, MN 55455, USA; abrah023@umn.edu; 5 ExoMed Diagnostic, Minneapolis, MN 55404, USA; nurtensaydam@yahoo.com

**Keywords:** OTX2, mTORC2, LMD, group 3 medulloblastoma

## Abstract

Medulloblastoma (MB) encompasses diverse subgroups, and leptomeningeal disease/metastasis (LMD) plays a substantial role in associated fatalities. Despite extensive exploration of canonical genes in MB, the molecular mechanisms underlying LMD and the involvement of the orthodenticle homeobox 2 (OTX2) gene, a key driver in aggressive MB Group 3, remain insufficiently understood. Recognizing OTX2’s pivotal role, we investigated its potential as a catalyst for aggressive cellular behaviors, including migration, invasion, and metastasis. OTX2 overexpression heightened cell growth, motility, and polarization in Group 3 MB cells. Orthotopic implantation of OTX2-overexpressing cells in mice led to reduced median survival, accompanied by the development of spinal cord and brain metastases. Mechanistically, OTX2 acted as a transcriptional activator of the Mechanistic Target of Rapamycin (mTOR) gene’s promoter and the mTORC2 signaling pathway, correlating with upregulated downstream genes that orchestrate cell motility and migration. Knockdown of mTOR mRNA mitigated OTX2-mediated enhancements in cell motility and polarization. Analysis of human MB tumor samples (N = 952) revealed a positive correlation between OTX2 and mTOR mRNA expression, emphasizing the clinical significance of OTX2’s role in the mTORC2 pathway. Our results reveal that OTX2 governs the mTORC2 signaling pathway, instigating LMD in Group 3 MBs and offering insights into potential therapeutic avenues through mTORC2 inhibition.

## 1. Introduction

Medulloblastoma (MB) stands as the predominant malignant brain tumor among children, constituting around 10–15% of all pediatric brain tumors [1,2,3]. Histologically, MBs are classified into various subtypes: classical, desmoplastic/nodular, extensive nodular, anaplastic, and large-cell MBs [4,5,6]. Genetically, MBs are subgrouped as WNT, SHH, Group 3, and Group 4. The WNT subgroup, comprising roughly 10% of all MBs, is characterized by CTNNB1 mutations, resulting in β-catenin accumulation within the nucleus [7]. Common driver genes include DDX3X, SMARCA4, TP53, and KMT2D3. Patients with the WNT subtype exhibit the most favorable prognosis, boasting a 5-year overall survival of 90% [8]. SHH MBs present an intermediate prognosis, with a 5-year overall survival of 60–80% [9]. These tumors exhibit genetic mutations in *PTCH1* or *SUFU* genes, both acting as negative regulators of the SHH signaling pathway [2,10]. MYCN and GLI2 copy number aberrations are characteristic of this subgroup [10,11]. Group 3 MBs, marked by high-level amplifications of transcription factor orthodenticle homeobox 2 (OTX2) and proto-oncogene c-MYC, are frequently associated with elevated OTX2 and c-MYC expression, leading to genomic instability and the poorest prognosis among all subgroups [10,12,13,14,15,16]. Group 4 MBs hold an intermediate prognosis. Amplifications of OTX2, MYCN, and CDK6 proto-oncogenes are commonly observed in this subgroup [10,11,17].

In 20–30% of all MB cases, metastatic disease has occurred at diagnosis and is associated with poorer prognosis [3,18]. MB metastatic patterns are subgroup-specific; metastatic dissemination is relatively rare in WNT and SHH MBs; conversely, Group 3 and 4 tumors are highly metastatic, particularly Group 3 tumors, which frequently display metastatic dissemination at first presentation or recurrence and are linked to poor prognosis [19,20]. Deep learning analysis of MB tumors has identified enriched RAS/MAPK pathway components in metastatic tumors [21]. Platelet-derived growth factor receptor (PDGFR) ERBB2 (HER2/neu) and receptors of tyrosine kinases (RTKs) upstream of RAS/MAPK have each been specifically implicated in MB pathogenesis and migration [22,23]. ERBB2, particularly when it is overexpressed in MB cell lines, significantly increases in vitro invasion and cell migration [24,25]. It also upregulates the expression of downstream RAS/MAPK pathway genes and S100A4, an S100 calcium-binding protein A4, which is implicated in the regulation of motility and tubulin polymerization [26]. In primary and metastatic compartments, PDGFRA has been identified at high levels of expression, suggesting that this gene is crucial in the maintenance of disease in both primary and metastatic compartments and implying it as a potential metastatic driver [27]. In fact, phosphorylation of RAS/MAPK downstream effectors (MAP2K1, MAP2K2, and MAPK1/3) is inhibited in MB cell lines treated with anti-PDGFRA, impairing cell motility and invasion [23]. Likewise, the PI3K/AKT pathway is overexpressed, suggesting a role in MB cell migration [28]. The pathway can be activated because the PTEN tumor suppressor is located on chromosome 10q, which is frequently deleted in MB [29,30,31]. In addition, Sleeping Beauty functional analysis has identified insertions in *PTEN*, *AKT2*, *PIK3R1*, *ARHGAP36*, and *FOXR2* genes that are implicated in MB dissemination [30,31,32,33,34]. Studies have highlighted NOTCH1’s role in MB metastasis initiation and self-renewal in Group 3 MBs [35]. In SHH-induced mouse models, several candidates such as ERAS, LHX1, AKT, ARNT, and GDI2 have been proposed as drivers of leptomeningeal dissemination [34,36,37]. However, these potential candidate genes’ relevance to leptomeningeal disease/metastasis (LMD) in human MB tumor tissues requires more extensive validation. Thus, it is imperative that the current understanding of the molecular drivers of MB dissemination leads to the identification of new therapeutic targets for the prevention and treatment of medulloblastoma leptomeningeal dissemination.

Extensive research has been conducted on the canonical driver genes in MB and the comprehension of the molecular mechanisms driving LMD. Despite this, the understanding of the potential involvement of the *OTX2* gene in LMD development remains significantly limited [38]. Recognizing the pivotal role of the *OTX2* gene as one of the major canonical driver genes of aggressive MB subgroup 3, we investigated the possible contributions of the *OTX2* gene on MB tumor growth and LMD. We found that overexpression of the *OTX2* gene resulted in increased cell growth, enhanced motility, and polarization of Group 3 MB cells. Subsequently, implanting these OTX2-overexpressing cells into mice resulted in reduced median survival compared to the control group, along with the development of spinal cord and brain metastases in a Cell-Derived Orthotopic Xenograft (CDOX) mouse model. Our investigation into the molecular mechanism underlying OTX2’s influence on metastasis revealed that the *OTX2* gene serves as a transcriptional activator of the Mechanistic Target of Rapamycin (mTOR) gene’s promoter and the mTOR Complex 2 (mTORC2) signaling pathway, correlating with the upregulation of downstream genes known to orchestrate cell motility and migration. Furthermore, siRNA-mediated knockdown of mTOR mRNA significantly reduced OTX2-mediated enhancements in cell motility and polarization. Moreover, through in-depth exploration and in silico analysis of human MB tumor samples, we found a positive correlation between OTX2 and mTOR mRNA expression in three independent MB datasets (N = 952), emphasizing the clinical significance of OTX2’s regulatory impact on the mTORC2 pathway. In addition, the treatment of MB cells with mTOR inhibitors resulted in a significant induction of cell death. Thus, our results collectively reveal that OTX2 regulates the mTORC2 signaling pathway and induces LMD in Group 3 MBs.

## 2. Results

**Figure 1:**
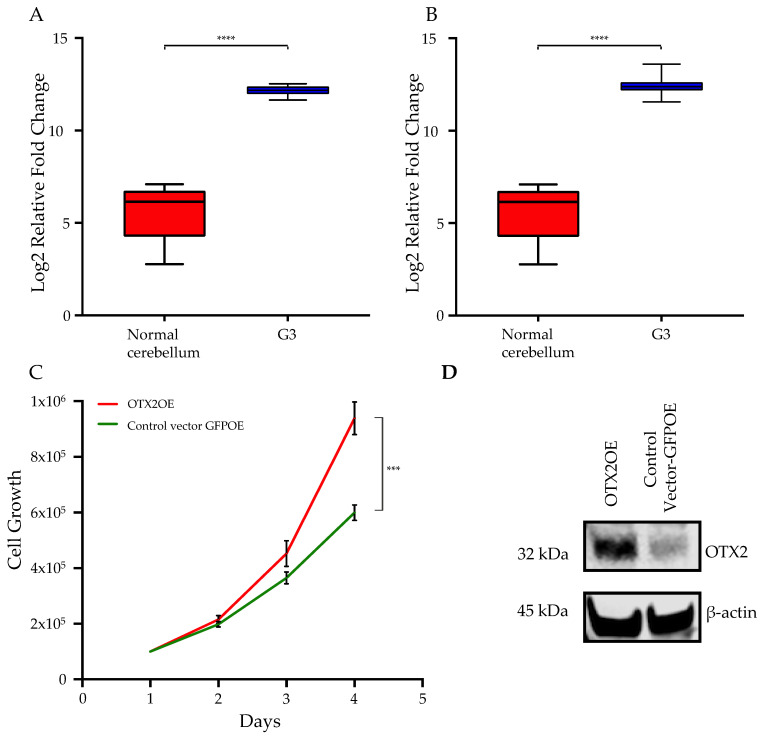
The *OTX2* gene is overexpressed in Group 3 medulloblastoma tumor samples, and triggers cell growth. *OTX2* gene expression was analyzed in two independent human medulloblastoma datasets (**A**) Robinson (N = 25), and (**B**) Northcott (N = 65) compared to normal cerebellum. Bioinformatics analysis was performed using R2: Genomics Analysis and Visualization Platform (http://r2.amc.nl). (**C**) 1 × 10^5^ D425 cells, transduced either with the OTX2 overexpression (OTX2OE) or the control GFP overexpression (GFPOE) lentiviral vectors, were plated and monitored for cell growth over the indicated days. Two biologically independent experiments are shown. (**D**) Verification of stable OTX2 overexpression in D425 cells is shown by Western blot using equal amounts of soluble whole-cell lysates.The original blot is shown as Supplementary Figure S1. β-actin served as the loading control. *** *p* < 0.0005, **** *p* < 0.00005 (Student’s *t*-test).

Exploiting recent large data libraries, we analyzed the gene expression status of the *OTX2* gene in two independent human MB datasets (N = 90) compared to that of normal cerebellum and found that the *OTX2* gene is overexpressed in Group 3 MB tumor tissues [10,39]. Bioinformatic analysis was performed using R2: Genomics Analysis and Visualization Platform on 15 November 2022 (http://r2.amc.nl) (Figure 1A,B). The finding that OTX2 is overexpressed in Group 3 MB tumor tissues aligns with previous research indicating the involvement of OTX2 as a key regulatory gene in these specific subgroups of MB [12,40]. This finding of heightened OTX2 expression in Group 3 MB samples contributes to our understanding of the molecular landscape of MB subtypes and emphasizes the potential importance of OTX2 as a driver gene in this specific subgroup.

### 2.1. Overexpression of the OTX2 Gene Increases Cell Motility and Cell Growth

**Figure 2:**
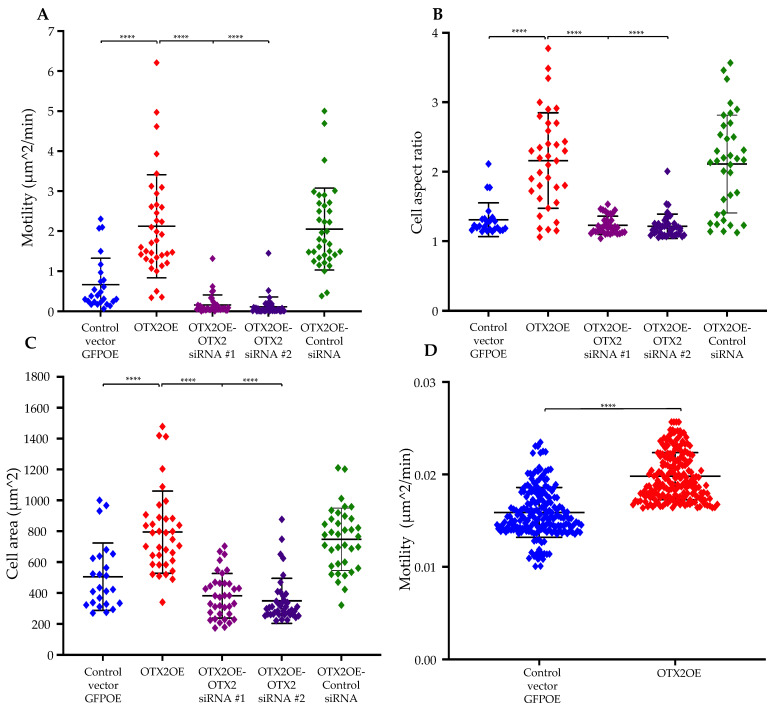
The *OTX2* gene overexpression enhances motility and polarization of D425 cells. Migration of D425 cells transduced either with the control vector (GFPOE) or OTX2 overexpression vector (OTX2OE) and transfected either with OTX2 or control siRNAs. Cells were placed on 4.6 kPa PAGs coated with type I collagen. Cells were monitored in a Nikon enclave TiE2 microscope and recorded time-lapse with intervals of 10 min for 16 h. The random motility coefficient (**A**), and cell aspect ratio (**B**) and cell area (**C**) of twenty to thirty individual cells per group were calculated using a customized MatLab script. D425 cells transduced either with the GFPOE or OTX2OE vectors were co-cultured with mouse cerebellum slices and recorded time-lapse with intervals Δt of 10 min for 24 h in a Zeiss LSM 7 Live swept-field laser confocal microscope. The random motility coefficient (**D**) of 400 individual cells (200 OTX2OE and 200 control vector GFPOE) is shown. Two independent siRNAs directed against the OTX2 mRNA were used. Error bars indicate means ± SD, and asterisks indicate significance compared to control vector GFPOE **** *p* < 0.00005 (Student’s *t*-test).

We used two human MB patient-derived Group 3-relevant cell lines, D425 and D341, and transduced these cell lines with an *OTX2* gene-carrying lentivirus vector. OTX2 overexpression significantly increased the growth of the MB cells compared to that of the control GFPOE-vector-transduced cells (Figure 1C; D425 cell line and Supplementary Figure S2; D341 cell line). Protein expression was confirmed by Western blot analysis (Figure 1D). We subsequently delved into exploring the potential impacts of OTX2 on MB cell mobility. To emulate the “in vivo” characteristics of the extracellular matrix, particularly its rigidity (a crucial factor influencing cell motility), we cultured MB cells overexpressing OTX2 (OTX2OE) or GFP (GFPOE) on a 4.6 kPa polyacrylamide hydrogel (PAG) coated with collagen type I. Human malignant brain tissue has been reported to exhibit an average stiffness of 4.6 kPa [41,42,43]. Tracking the trajectories of randomly chosen cells over a 16 h period, we utilized a Nikon Ti2 microscope to observe and record cell migration and morphology. Employing an image segmentation MATLAB algorithm, we quantified cell position, spread area, and aspect ratio. The mean squared displacement for each cell enabled the estimation of cell random motility coefficients. In D425 cells overexpressing the *OTX2* gene, we observed significantly heightened motility, cell aspect ratio, and cell area compared to cells carrying the control GFPOE vector (RMC 2.12263 μm^2^/min vs. 0.665572 μm^2^/min, *p* < 0.00001; 2.1607 vs. 1.30822, *p* < 0.0001; 794.2 μm^2^ vs. 505.677 μm^2^, *p* < 0.001) (Figure 2A–C). To confirm the role of OTX2 in driving cell motility in MB, we knocked down OTX2 mRNA in D425 overexpression cells using two small interfering RNAs (siRNAs). Following OTX2 knockdown, D425-OTX2OE exhibited a significant decrease in cell motility, cell aspect ratio, and cell area (0.162892 μm^2^/min vs. 0.665572 μm^2^/min, *p* < 0.0001; 1.22858 vs. 1.30822, *p* < 0.01; 328.33 μm ^2^ vs. 505.677 μm^2^, *p* < 0.001) compared to the control siRNAs. (Figure 2A–C). In addition, the OTX2 silencing in D341-OTX2OE cells using two different siRNAs revealed a prominent inhibition in cell motility compared to control siRNA-transfected cells (RMC 2.3993 μm^2^/min vs. siRNA#1: 0.145397 μm^2^/min/siRNA#2: 0.352627 μm^2^/min, *p* < 0.0001; siRNA#1: 0.145397 μm^2^/min/siRNA#2: 0.352627 μm^2^/min vs. 0.975558 μm^2^/min, *p* < 0.001) (Supplementary Figure S3). Likewise, cell aspect ratio and cell area were highly decreased in D341-OTX2OE cells transfected with OTX2 siRNA (2.36473 vs. siRNA#1: 1.62432/siRNA#2: 1.62702, *p* < 0.0001; 1401.55 μm^2^ vs. siRNA#1: 993.901 μm^2^/siRNA:2: 937.0387 μm^2^, *p* < 0.0001) (Supplementary Figure S3). These results demonstrate a novel role of the *OTX2* gene in leading migration in Group 3 MB cells. Recognizing that MB develops in the cerebellum, we further simulated the tumor microenvironment of MBs by preparing mouse cerebellar slices for an ex vivo cell migration experiment. As illustrated in Figure 2D, under these experimental conditions, D425-OTX2OE cells exhibited significantly increased motility compared to control cells (Figure 2D). These findings indicate that overexpression of OTX2 enhances cell motility in MB, suggesting a potential role for OTX2 in driving migratory behavior within the tumor microenvironment.

### 2.2. The OTX2 Gene Induces MB Tumor Growth and Triggers Spinal Cord/Brain Metastases in Cell-Derived Orthotopic Xenograft (CDOX) Mouse Model

**Figure 3:**
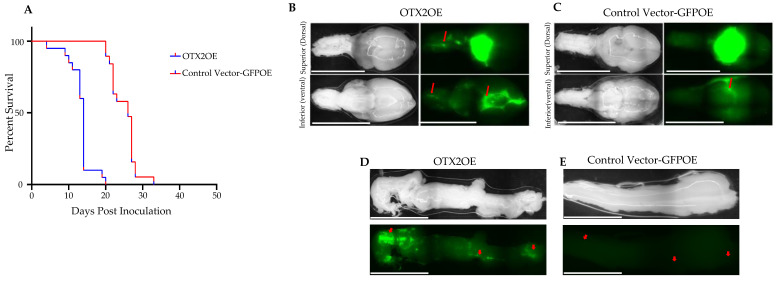
Overexpression of the *OTX2* gene induces brain and spinal cord metastases in Group 3 MB. (**A**) Kaplan–Meier curves depicting overall survival of mice injected either with control vector-GFPOE (N = 19) and OTX2 overexpressing (OTX2OE) D425 cells (N = 20). Cells were implanted by stereotactic injection into the cisterna magna of nude mice. Mice were anaesthetized and perfused with 1XPBS, and the brain (**B**,**C**) and spinal cords (**D**,**E**) were collected and examined for GFP protein expression under a Nikon AZ100 C1SI spectral confocal microscope. Representative images are shown. White lines on the bright field are caused by the light reflection. The spinal cord from (**D**) OTX2OE or (**E**) control vector GFPOE bearing mice was also collected and analyzed for GFP expression. Representative images are shown. White lines on the bright field are caused by the light reflection. Red arrows point to the brain and spinal cord metastases or lack thereof. Scale 5000 μm. *p*-values were determined using the log-rank method, *p* = 0.00014.

We further investigated whether the observed differences in migration rate and morphology correlated with disease progression and survival, given the increased motility of D425-OTX2OE cells compared to D425-GFPOE cells. To address this inquiry, we orthotopically implanted either D425-GFPOE or D425-OTX2OE cells into the cisterna magna of immune-deficient nude mice and assessed the survival times of the tumor-bearing mice. Our findings revealed that the OTX2OE cohort exhibited a significantly lower median survival than the GFPOE control cohort (OTX2OE N = 20, GFPOE N = 19, 20 days vs. 33 days, log-rank test, *p* < 0.00014; Figure 3A). Further examination of the brains from both cohorts, analyzing GFP fluorescence expression, revealed that OTX2OE cells metastasized more frequently to both the dorsal and ventral regions of the brains compared to the control group (Figure 3B,C). Notably, the ventral brain of mice grafted with OTX2OE cells displayed multiple aggregates of MB cells, a phenomenon less prominent in the GFPOE control mice (Figure 3B,C). Additionally, upon removal of the spinal cord from both cohorts, it was evident that D425-OTX2OE cells exhibited significantly greater dissemination into the spinal cord compared to the mice implanted with GFPOE cells (Figure 3D,E). Collectively, these findings strongly suggest that OTX2 plays a pivotal role in promoting the metastatic behavior of Group 3 MB cells in mice. Understanding the mechanisms by which OTX2 drives leptomeningeal metastases to the brain and spinal cord could unveil critical pathways or targets for interventions aimed at inhibiting metastatic progression in this aggressive form of MB.

### 2.3. OTX2 Gene Upregulates the mTORC2 Signaling Pathway

**Figure 4:**
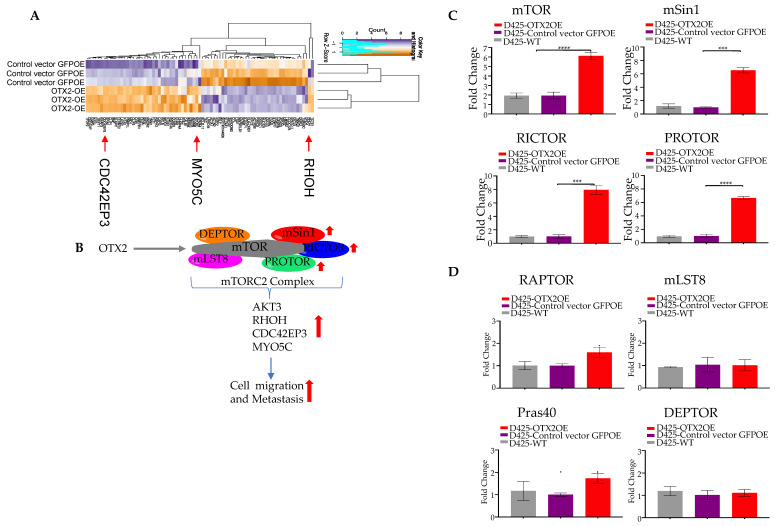
The *OTX2* gene activates the mTORC2 pathway: (**A**) Heatmap showing mTORC2 genes regulated by *OTX2* gene from an RNA sequencing data performed in triplicate, (**B**) Schematic diagram of OTX2/mTORC2 pathway interaction is shown. The transcript levels of mTORC2, (**C**) and mTORC1 genes (**D**) were assessed by qRT-PCR and normalized to the housekeeping gene hb-actin. Statistical significance was determined using Student’s *t*-test (*** *p* < 0.0005, **** *p* < 0.00005). Error bars indicate means ± SD. Two independent experiments were carried out, and three replicates were performed. Arrows indicate activation.

**Figure 5:**
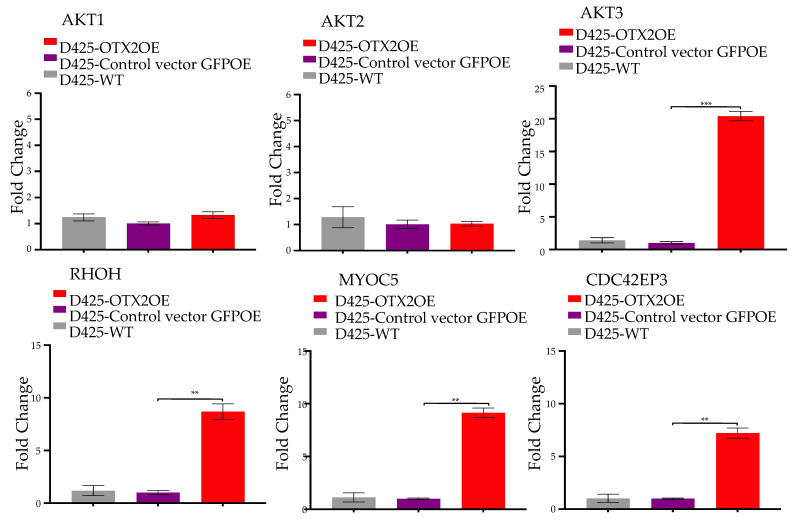
qRT-PCR validation of migratory genes regulated by *OTX2* gene expression. To confirm the migratory genes identified through bulk RNA sequencing, we utilized the ΔΔCq method to quantify the augmented mRNA expression of downstream genes associated with mTORC2. The obtained values were normalized to the housekeeping gene hb-actin. Statistical significance was determined using Student’s *t*-test (** *p* < 0.005, *** *p* < 0.0005). Error bars indicate means ± SD. Two independent experiments were carried out, and three replicates were performed.

Our investigation into the molecular mechanisms underlying OTX2’s impact on metastasis in Group 3 MB involved a comprehensive analysis using bulk RNA sequencing. This analysis identified 1171 statistically significant dysregulated genes specific to *OTX2* gene overexpression, demonstrating a fold change greater than 2 and a *p*-value less than 0.05 compared to control GFPOE cells. Among these genes, 739 were upregulated and 432 were downregulated. Gene set enrichment analysis revealed a significant enrichment of the mTOR signaling gene set related to cell migration in OTX2-overexpressing cells compared to control cells transduced with the GFPOE vector. Furthermore, this activation of mTOR signaling correlated with the upregulation of downstream genes known to orchestrate cell motility and migration, including RHOH, MYOC5, and CDC42EP3, in the Group 3 D425 cells (Figure 4A, Table S1). mTOR is a serine/threonine protein kinase crucial in regulating cellular processes such as cell growth, proliferation, survival, and migration. It is part of two distinct multi-protein complexes: mTOR Complex 1 (mTORC1) and mTORC2. mTORC2 specifically regulates cell survival, cytoskeletal organization, and migration, playing a pivotal role in the activation of AKT and other kinases. Given these findings, we hypothesized that OTX2 overexpression activates the mTORC2 complex and its downstream effector proteins, such as RHOH, MYOC5, CDC42EP3, and AKT3, which are involved in cell survival and migration. Our working model is summarized in Figure 4B. To test our hypothesis, we conducted several qRT-PCR experiments and found that the mTORC2 partners, mSin1, RICTOR, and PROTOR, were significantly upregulated (Figure 4C), while mTORC1 partners, RAPTOR, mLST8, and Pras40, were not found to be significantly dysregulated (Figure 4D) in OTX2 OE cells compared to control cells. This suggests that the mTORC2 signaling pathway is specifically regulated by *OTX2* gene expression. Additionally, we validated the upregulation of downstream migratory genes (AKT3, RHOH, MYOC5, and CDC42EP3) by qRT-PCR in D425-OTX2OE cells compared to the control (Figure 5).

### 2.4. OTX2 Gene Transcriptionally Activates the mTOR Promoter

**Figure 6:**
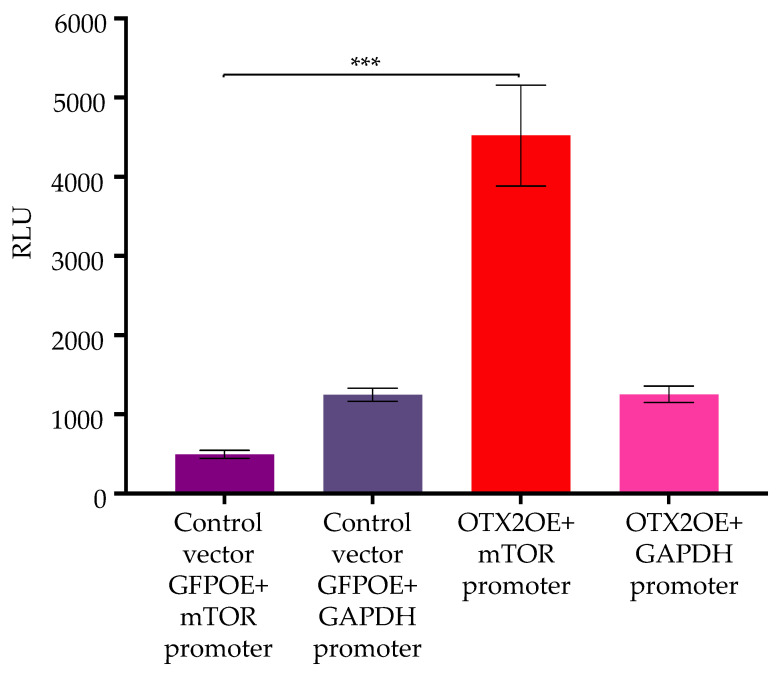
The *OTX2* gene transcriptionally activates mTOR gene’s promoter. Culture medium from cells transfected either with mTOR promoter-LUC+ or GAPDH-LUC+ reporter clones was collected. Utilizing 30 μL per assay, luciferase activity was assessed via the Pierce Gaussia Luciferase Glow Assay Kit and quantified with a luminometer (Synergy Biotek). Two independent experiments were conducted, and three biological replicates per treatment were performed per each one. Error bars represent means ± SD, with asterisks denoting significance compared to the control vector GFPOE + mTOR promoter (*** *p* < 0.0005, Student’s *t*-test). All analyses were performed using GraphPad Prism 9 Software.

To investigate the molecular pathways through which the *OTX2* gene modulates the mTORC pathway, particularly the impact of the OTX2 oncogene on mTOR transcriptional levels, we utilized a construct incorporating the Gaussia Luciferase gene under the regulation of the mTOR promoter (HPRM45818-PG02, GeneCopoeia). Following transfection of D425-OTX2OE or D425-GFPOE cells with this plasmid and a subsequent 48 h interval, luciferase activity was quantified. Our findings, depicted in Figure 6, exhibited notably elevated luciferase activity in OTX2-overexpressing cells (D425-OTX2OE) in comparison to the control GFP-overexpressing cells (D425-GFPOE) transfected with the same promoter. These results strongly suggest that the *OTX2* gene exerts transcriptional activation on the mTOR gene’s promoter. To ensure experimental fidelity, a GAPDH promoter (GAPDH-PG02) was included as a control in these conditions.

### 2.5. siRNA-Mediated Knockdown of mTOR mRNA Significantly Reduced OTX2-Mediated Enhancements in Cell Motility and Polarization

**Figure 7:**
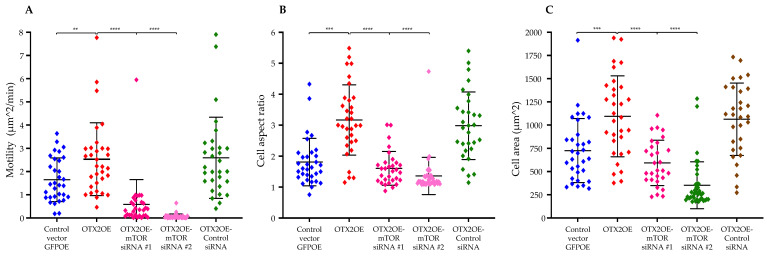
Knockdown of mTOR mRNA inhibits migration of D425 cells overexpressing the *OTX2* gene. Migration of D425 cells transduced either with the OTX2 overexpression plasmid (OTX2OE) or control vector GFPOE and transfected with mTOR or control siRNAs are presented. After 48 h of transfection cells were placed on 4.6 kPa PAGs coated with type I collagen. Cells were monitored in a Nikon Ti2 microscope and recorded time-lapse with intervals of 10 min for 16 h. The random motility coefficient (**A**), cell aspect ration (**B**), and cell area (**C**) of thirty individual cells per treatment were calculated using a customized MatLab script. This experiment was repeated two times. Two independent siRNAs directed against the mTOR mRNA were used. Error bars indicate means ± SD, and asterisks indicate significance compared to the control vector GFPOE. ** *p* < 0.005, *** *p* < 0.0005, **** *p* < 0.00005 (Student’s *t*-test).

To further elucidate the migratory mechanism influenced by the *OTX2* gene and its connection with mTOR signaling, we utilized siRNA technology to specifically target and knock down mTOR mRNA expression. Subsequent migration assays conducted with PAG revealed a significant decrease in migratory potential in D425-OTX2OE cells transfected with mTOR siRNAs compared to control siRNA-transfected cells (RMC 2.53197 μm^2^/min vs. siRNA#1: 0.58708 μm^2^/min/siRNA#2: 0.064596; *p* < 0.0001). Additionally, D425-OTX2OE cells exhibited an increase in cell aspect ratio and cell area (3.16742 vs. siRNA#1: 1.60687/siRNA#2: 1.36071; *p* < 0.0001; 1094.79 μm^2^ vs. siRNa#1: 593.142 μm^2^/siRNA#2: 352.498; *p* < 0.0001) (Figure 7). Under these experimental conditions, we observed a significant reduction in the mRNA level of mTOR by approximately 80–85% as assessed by qPCR (Supplementary Figure S4).

Similarly, silencing mTOR in D341-OTX2OE cells dramatically decreased migration compared to control siRNA-transfected cells and D341-GFPOE (RMC 2.15524 μm^2^/min vs. 0.104613 μm^2^/min, *p* < 0.0001; 1.02964 μm^2^/min vs. 0.104613 μm^2^/min, *p* < 0.00001). In addition, siRNA-transfected D341-OTX2OE cells displayed decreased cell aspect ratio (1.77163 vs. siRNA#1: 1.40717/siRNA#2: 1.29585, *p* < 0.0001; 1.36167 vs. sirRNA#1: 1.40717/siRNA#2: 1.29585, *p* < 0.0001) and cell area (1249.36 μm^2^ vs. siRNA#1: 901.293 μm^2^/siRNA#2: 924.492 μm^2^, *p* < 0.001; 1020.59 μm^2^ vs. siRNA#1: 901.293 μm^2^/siRNA#2: 924.492 μm^2^, *p* < 0.001) (Supplementary Figure S5). Collectively, these findings strongly suggest that OTX2 regulates MB cell motility through the upregulation of the mTORC2 complex.

### 2.6. Clinical Relevance of Gene Expression Analysis and Significant Positive Correlation between OTX2 and mTOR mRNAs in Human MB Tumor Samples

**Figure 8:**
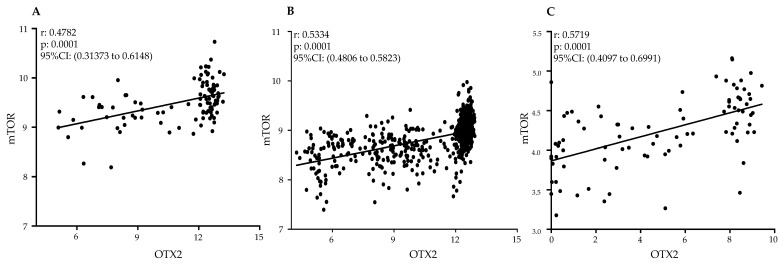
Clinical Relevance of Gene Expression Analysis of OTX2 and mTOR mRNAs. Positive associations between *OTX2* and *mTOR* gene expression were observed in primary medulloblastoma tumor samples. Pearson correlation coefficients were calculated to quantify the level of association between OTX2 and mTOR expression using three independent datasets: (**A**) Northcott (N = 103), (**B**) Cavalli (N = 763), and (**C**) Okonechnikov (N = 86). Regression lines were generated for each dataset to visually represent the correlation trends. All analyses were performed using GraphPad Prism 9 Software.

In order to explore the presence of the OTX2-mTOR signaling pathway interaction in human MB tumors, we conducted an extensive in silico data analysis and found a significant positive correlation between OTX2 and mTOR mRNA expressions across a substantial dataset of human tumor samples (n = 952) obtained from three independent datasets, as depicted in Figure 8: Northcott dataset (N = 103) [44], the Cavalli dataset (N = 763) [16], and the Okonechnikov dataset (N = 86) [45]. The robust consistency of this correlation across diverse datasets strongly supports the validity of our findings. This compelling evidence underscores the clinical relevance of our discovery concerning the regulatory influence of OTX2 on the mTOR pathway in the context of MB.

### 2.7. The Treatment of MB Cells with mTOR Inhibitors Resulted in a Significant Induction of Cell Death

**Figure 9:**
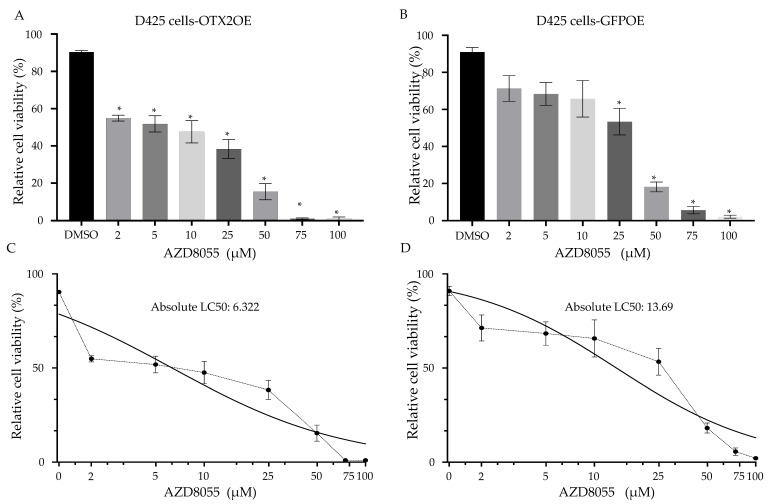
Rapamycin (AZD8055) inhibits cell viability of D425 MB cells. Effect of 48-h treatment of AZD8055 at indicated concentrations on cell viability of (**A**) D425-OTX2OE cells, and (**B**) D425-GFPOE cells. Cells were seeded in a 24 well plate at a density of 5 × 10^4^ cells/well 24-h prior to treatment the indicated concentrations of rapamycin (AZD8055). 48-h after treatment, cells were stained with trypan blue (0.4%, Invitrogen), and then counted by using a Neubauer cell counting chamber. Each plotted value represents the mean percentage of live cells per well. LC50 values were calculated (**C**) OTX2OE and (**D**) control GFP cells. Error bars indicate ± SD. * *p* < 0.05, Student’s *t*-test (DMSO compared to treatment). Experiment was performed in triplicate.

**Figure 10:**
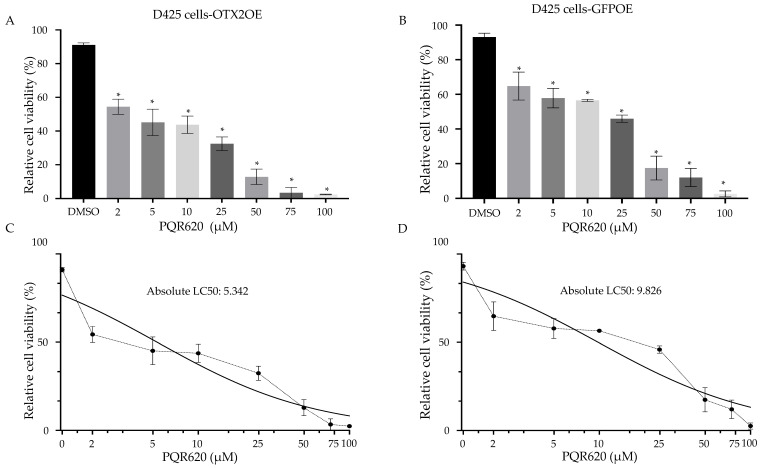
Rapamycin (PQR620) inhibits cell viability of D425 MB cells. Effect of 48-h treatment of PQR620 at indicated concentrations on cell viability of (**A**) D425-OTX2OE cells, and (**B**) D425-GFPOE cells. Cells were seeded in a 24 well plate at a density of 5 × 10^4^ cells/well 24-h prior to treatment the indicated concentrations of rapamycin (PQR620). 48-h after treatment, cells were stained with trypan blue (0.4%, Invitrogen), and then counted by using a Neubauer cell counting chamber. Each plotted value represents the mean percentage of live cells per well. LC50 values were calculated (**C**) OTX2OE and (**D**) control GFPOE cells. Error bars indicate ± SD. * *p* < 0.05, Student’s *t*-test (DMSO compared to treatment). Experiment was performed in triplicate.

To explore potential therapeutic avenues, we tested two commercially available mTOR inhibitors, AZD8055 and PQR620, that can pass through the blood–brain barrier (BBB) [46,47,48]. Both compounds significantly inhibited the growth of D425 cells compared to control solvent-treated cells. (AZD8055: Figure 9 and PQR620: Figure 10). As demonstrated in Figure 9C,D and Figure 10C,D, the IC50 doses of both drugs were lower in OTX2-overexpressing cells compared to control GFP-transduced cells. This suggests that OTX2 overexpression rendered these cells more susceptible to rapamycin treatment. Collectively, our findings suggest that mTORC2 is directly involved in the cell growth and/or migration mediated by *OTX2* gene expression, highlighting the therapeutic potential of targeting mTORC2 in Group 3 MB.

## 3. Discussion

The poor prognosis and frequently fatal outcome of Group 3 MBs are attributed to the fact that MB cells metastasize to the leptomeningeal space. Regrettably, the mechanisms behind the metastasis of MB remain largely unknown, and research recognizing the role of OTX2 in MB has mainly focused on the epigenetic and transcriptional activation of cell cycle genes and tumor growth. Here, we have shown the unique role of OTX2 in driving metastasis in Group 3 MBs. Our study unraveled the multifaceted role of the *OTX2* gene in driving aggressive behaviors in Group 3 MBs. Analysis of human MB datasets revealed heightened OTX2 expression in these specific tumor subtypes, aligning with previous indications of OTX2’s regulatory involvement in these groups [49,50,51]. This robust correlation emphasizes the potential significance of OTX2 as a therapeutic target in Group 3 MB, offering valuable insights into the molecular landscapes characterizing distinct MB subtypes. Notably, OTX2 overexpression significantly promoted MB cell growth, an effect observed through lentiviral vector transduction. The increase in cell growth was accompanied by Western blot-confirmed protein expression, confirming the role of OTX2 in fostering enhanced cellular proliferation within MB tumors.

Expanding the investigation to MB cell motility, our study discovered that *OTX2* gene overexpression distinctly heightened cell motility and morphological changes in vitro. This effect was evident across diverse experimental setups, including 4.6 kPa PAG cultures mimicking the tumor microenvironment and ex vivo mouse cerebellar slice assays [43,51]. These findings collectively highlight OTX2’s potential in driving migratory behaviors within the MB tumor microenvironment, implying a broader influence on tumor progression.

Further experiments using an orthotopic xenograft mouse model demonstrated a pronounced reduction in median survival and an increased occurrence of spinal cord and brain metastases in animals implanted with OTX2-overexpressing MB cells. The metastatic propensity of these cells indicates OTX2’s pivotal role in promoting aggressive behavior in Group 3 MBs, specifically influencing metastatic dissemination to critical neurological sites. Our results align with previous reports in which OTX2’s role in migration is highlighted. Ferrucci et al. reported that, in Group 3 tumors, the highly expressed gene PRUNE1 acts as a crucial molecular driver of metastasis by activating canonical TGF-β signaling through its binding to NME1. This activation leads to an increase in OTX2 expression and inhibition of PTEN, forming a signaling axis that promotes metastasis [52]. The complexity of LMD in MB calls for a deeper understanding of the mechanisms governing metastatic dissemination. In this regard, several metastatic drivers have been proposed. NOTCH1 emerges as another pivotal driver of metastasis initiation in Group 3 MBs, with TWIST1 and BMI1 serving as downstream mediators [35]. Intrathecal administration of a NOTCH1-blocking antibody to MB tumor-bearing mice results in a lower incidence of spinal metastasis compared to the control group. BMI1, corroborated in two independent studies, also plays a role in promoting MB invasion and metastasis [53].

Molecular investigations of underlying pathways revealed OTX2’s activation of the mTOR signaling pathway. Transcriptomic analyses identified a suite of genes associated with increased cell motility, providing crucial insights into the downstream effects orchestrated by OTX2. The findings indicated a specific association between OTX2 and the mTORC2 complex, correlating with the upregulation of downstream genes implicated in cell migration and survival. Targeting the OTX2-mediated mTOR pathway emerged as a potential therapeutic strategy. In silico analysis of human MB tumor samples reinforced the clinical relevance, revealing a robust positive correlation between OTX2 and mTOR mRNA expressions across diverse datasets. Moreover, treatment with mTOR inhibitors induced significant cell death in OTX2-overexpressing MB cells, suggesting the therapeutic potential of targeting mTOR in Group 3 MBs. The complexity of LMD in MB calls for a deeper understanding of the mechanisms governing metastatic dissemination. While other studies offer tantalizing insights into potential drivers of LMD, the lack of widespread validation in human tumor tissue underscores the gap in our understanding [34,36,37,38]. Elucidating the molecular underpinnings of LMD in human MB is crucial for developing targeted therapies aimed at inhibiting metastatic progression and improving patient outcomes.

In summary, these comprehensive findings underscore OTX2’s multifaceted role in driving aggressive behavior within specific MB subtypes. Our study illuminates the paramount role of the *OTX2* gene in driving metastatic behavior in Group 3 MBs. Through various analyses and experimental models, we unveil OTX2’s multifaceted influence, showing its association with heightened cell growth, increased migratory behaviors within the tumor microenvironment, and promotion of aggressive behavior in MB. Further molecular investigations uncover OTX2’s orchestration of the mTORC2 signaling pathway, correlating with enhanced cell motility and survival. This study highlights OTX2 as a key player in MB metastasis. Our findings offer potential therapeutic avenues by targeting the OTX2-mediated mTORC2 pathway in order to mitigate metastatic progression in these aggressive MB subtypes. Targeting the OTX2-mediated mTORC2 pathway emerges as a potential therapeutic strategy, holding significant promise in managing these aggressive tumors.

Our findings regarding the role of OTX2 in driving aggressive behaviors and metastasis in Group 3 MBs also have several potential applications in drug discovery and development: (I) Target Identification: Our study identifies OTX2 and the mTORC2 pathway as key players in promoting metastasis in Group 3 MBs. This provides drug developers with specific targets to focus on when designing new therapies. (II) Drug Screening: Our study suggests that targeting OTX2 or the mTORC2 pathway could be effective in inhibiting metastatic progression. Drug discovery efforts can involve screening libraries of compounds to identify potential inhibitors or modulators of these targets. (III) Therapeutic Development: Based on our findings, researchers can develop drugs that directly target OTX2 or the mTORC2 pathway. These could include small-molecule inhibitors, monoclonal antibodies, or gene therapy approaches aimed at disrupting the signaling pathways associated with aggressive MB behavior. (IV) Preclinical Testing: Drugs targeting OTX2 or the mTORC2 pathway can undergo rigorous preclinical testing using in vitro models, animal models, and patient-derived xenografts to assess efficacy, safety, and pharmacokinetics before advancing to clinical trials. (V) Clinical Trials: Translating preclinical findings into clinical practice involves conducting clinical trials to evaluate the safety and efficacy of novel therapies targeting OTX2 or the mTORC2 pathway in human MB patients. These trials can help validate the therapeutic potential identified in preclinical studies and guide treatment protocols.

In summary, our study’s insights into OTX2-mediated metastasis provide valuable opportunities for drug discovery and development, ranging from target identification and drug screening to therapeutic development, biomarker identification, and clinical testing. These efforts have the potential to significantly impact the management and outcomes of patients with aggressive Group 3 MBs.

Limitations of this study: Despite shedding light on the pivotal role of the *OTX2* gene in MB aggressiveness, this study predominantly focused on in vitro and murine models. An inherent limitation of this study is the absence of direct mTOR inhibitor investigations in human MB patients. While our findings demonstrate promising outcomes in cell and animal models, translating these results to clinical settings and evaluating the efficacy and safety of mTOR inhibitors in patients remains unexplored. Conducting clinical trials to assess the therapeutic potential of mTOR inhibitors in human MB is essential for validating the translational relevance of our preclinical findings.

## 4. Materials and Methods

### 4.1. Cell Lines

D341 (ATCC^®^ HTB-187TM, RRID:CVCL_0018) and HEK293T (ATCC^®^ CRL-3216, RRID:CVCL_0063) were purchased from ATCC, and D425 (SCC290, RRID:CVCL_1275) was purchased from Sigma-Millipore (Sigma-Aldrich, St. Louis, MO, USA) [54,55,56]. Human medulloblastoma cells were grown in Minimum Essential Media (MEM; Gibco 11095; Thermo Fisher Scientific, Waltham, MA, USA) plus 15% fetal bovine serum (FBS; Gibco 26140079; Thermo Fisher Scientific, Waltham, MA, USA), 100 mg/mL penicillin G, and 100 lg/mL streptomycin (Gibco 15140122). HEK293T cells were grown in Dulbecco’s Modified Eagle’s Medium (DMEM; Gibco 11965092) supplemented with 10% FBS (Gibco 26140079), 100 mg/mL penicillin G, and 100 lg/mL streptomycin (Gibco 15140122; Thermo Fisher Scientific, Waltham, MA, USA). All cell lines were cultured at 37 °C in a humidified environment with 5% CO_2_ and confirmed mycoplasma negative (Lonza MycoAlert LT07-318; Basel, Switzerland) (most recently confirmed negative in January 2024).

### 4.2. Cerebellum Inoculation

Immunodeficient mice (Athymic nude, RRID:MGI:5652489) 6 to 8 weeks old were purchased from Charles River Laboratories. Cerebellar inoculation: Mice were anesthetized with an intraperitoneal injection of 0.03 mL ketamine HCl (100 mg/mL) (Dechra Pharmaceuticals 200-073; Northwich, England, UK)/xylazine (20 mg/mL) (Bimeda 61133-6017-1; Schaumburg, IL, USA). For the stereotactic intracranial injection, the surgical site was shaved and prepared with 70% ethyl alcohol (Decon Labs, INC 27010; King of Prussia, PA, USA). Mice were placed in a stereotactic frame and a midline incision in the back of the brain was made with a scalpel. MB cells (2 × 10^5^), D425OTX2OE, or D425 control vector GFPOE in 5 µL FBS (FBS; Gibco 16000044) were implanted by stereotactic injection into the cerebellum in accordance with the following stereotactic coordinates: lambda 2 mm right, 2 mm down, and 2 mm deep using a Hamilton syringe with a needle (Hamilton 80300) [57,58,59]. The needle was removed, and the skin was sutured using 4-0 nylon thread. Immediately after surgery, mice were injected with 10 mg/Kg Carprofen (Norbrook 515.00719.3; Newry, Northern Ireland, UK) and exposed to a heating system. In addition, mice were administered Carprofen 10 mg/Kg for three days following tumor inoculation [60]. Mice were sacrificed by CO_2_ euthanasia at neurological endpoints or when signs of the disease were displayed (no drinking, lethargic, hunched back, head tilt, weight loss >10% as endpoints). A Kaplan–Meier curve was used to estimate the survival of mice, and the statistical significance was evaluated using a log-rank test.

### 4.3. Metastasis Acquisition Using GFP Fluorescence

The whole brain and spinal cord were removed and analyzed for GFP fluorescence under direct observation using an AZ100M Macro fluorescence microscope (Nikon, Minato City, Tokyo, Japan). Per mouse, 7 to 10 dorsal and ventral images of the brain and spinal cord were acquired with consistent settings. All images were analyzed using Fiji/ImageJ (https://imagej.nih.gov/ij/download.html, accessed on 19 March 2024; RRID:SCR_003070) [61].

### 4.4. Lentiviral Infections and Transduction

Lentiviruses were produced “all-in-one” by cotransfection of HEK293T cells with 2 μg pLV(3)-EGFP:T2A:Puro-CMV>hOTX2[NM_001270525.1 (VectorBuilder Inc., Chicago, IL, USA) and packaging plasmids: 1 μg pCMV (Addgene, RRID:17452; Watertown, MA, USA) and 1 μg pMD2.G (Addgene, RRID:12259; Watertown, MA, USA). Transfections were carried out with the Dharmacon^TM^ Trans-Lentiviral packaging kit (Horizon Discovery, Cambridge, UK) according to the manual’s instructions. Lentiviral particles were harvested at 72 h post-transfection. Approximately 5 × 10^5^ D425 or D341 cells per 6-well plate were transduced with 2 mL of media containing lentivirus particles in the presence of 10 μg/mL of polybrene (Santa Cruz sc-134220A, Dallas, TX, USA) for 24 h. The next day, cells were selected with 1 μg/mL puromycin (InvivoGen ant-pr-1; San Diego, CA, USA) and continuously grown under puromycin selection [62,63,64]. A control GFPOE lentiviral vector was produced to use as a control. Transduced cells were sorted for GFP expression, and RT-qPCR and Western blot were performed to determine knockdown efficiency by assessing mRNA transcript levels and protein expression of OTX2.

### 4.5. RT-qPCR Assays

Total RNA from D425 cells was isolated using Trizol (Invitrogen 15596026; Thermo Fisher Scientific, Waltham, MA, USA), and cDNA was synthesized with the qScript cDNA Synthesis Kit (Quantabio 95047-100; Beverly, MA, USA) using 1000 ng of total RNA. cDNA was analyzed by quantitative PCR with the SYBR Green qPCR Mix (Quantabio 95053-500; Beverly, MA, USA). The input cDNA amount was 15 ng per reaction, and for each mRNA analyzed, four qPCR reactions were performed independently. qPCR conditions were 35 cycles and the following cycling conditions: 95 °C: 3 min; 90 °C: 1 min; 60 °C: 1 min; 72 °C: 1 min; 72 °C: 5 min. Relative expression analysis was performed using the ΔΔCq method. The comparative cycle threshold (Cq) values of the samples were normalized to the housekeeping gene, human B-actin, and to the control vector GFPOE [65,66,67]. Statistical significance was compared to the control vector GFPOE. Primers for qPCR were designed using primer-BLAST and based on the following Homo sapiens template sequences at the NCBI database: NM_001386500.1 for Mechanistic Target of Rapamycin kinase (MTOR), NM_001283012.2 for DEP domain-containing mTOR-interacting protein (DEPTOR), NM_001163034.2 for regulatory-associated protein of MTOR Complex 1 (RPTOR), NM_001098632.2 for AKT1 substrate 1 (AKT1S1) (PRAS40), NM_001199173.3 for MTOR-associated protein, LST8 homolog (MLST8), NM_001285439.2 for RPTOR-independent companion of MTOR Complex 2 (RICTOR), NM_001160167.2 for proline-rich 5-like (PRR5L) (Protor 2), NM_001006617.3 for MAPK-associated protein 1 (MAPKAP1) (mSIN1), NM_001014431.2 for AKT serine/threonine kinase 1 (AKT1), NM_001243027.3 for AKT serine/threonine kinase 2 (AKT2), NM_001206729.2 for AKT serine/threonine kinase 3 (AKT3), NM_001278359.2 for ras homolog family member H (RHOH), XM_047432845.1 for myosin VC (MYO5C), and NM_001270436.2 for CDC42 effector protein 3 (CDC42EP3). The set sequences are listed in Table S2.

### 4.6. Western Blot Analysis

Western blotting was performed as previously described [67,68,69]. For whole-cell lysate, the cell pellet was resuspended in homogenizing buffer (100 mL DI water (Gibco 10977015), 1.516 g TRIS (Sigma-Aldrich 102478929), and 100 µL of 0.1% Tween (Promega H5151) supplemented with a protease and phosphatase inhibitor (Thermo Fisher Scientific A32959; Waltham, MA, USA) and incubated for 30 min. The lysate was sonicated for ~5 s two times or until the tissue was fully lysed using a Sonifier 250 (Branson; Danbury, CT, USA). Protein concentration was quantified by Pierce^TM^ BCA Protein Assay Kit (Thermo Fisher Scientific 23227). Next, 30 µg of whole-cell lysate was electrophoresed on a Mini Protean Tris-glycine gel (Bio-Rad 4569034; Hercules, CA, USA) and subsequently transferred onto a 0.45 µm polyvinylidene difluoride membrane (Thermo Fisher Scientific 88585; Waltham, MA, USA). The membrane was incubated in blocking buffer (Rockland MB-070; Philadelphia, PA, USA) for an hour and was incubated overnight with primary anti-OTX2 (1:500 dilution, (D7Y3J) Rabbit mAb #11943, Cell signaling, RRID:AB_2797774; Danvers, MA, USA) and anti-B-actin (1:5000 dilution, (8H10D10) Mouse mAb #3700, Cell signaling, RRID:AB_2242334; Danvers, MA, USA). Then, the blot was kept in blocking buffer for an hour. The membrane was treated with two secondary antibodies, Alexa Fluor^®^ 790-AffiniPure Donkey Anti-Rabbit IgG (H+L) (Jackson ImmunoResearch Inc., RRID:AB_2922889; West Grove, PA, USA) and Alexa Fluor^®^ 680-AffiniPure Donkey Anti-Mouse IgG (H+L) (Jackson ImmunoResearch Inc., RRID:AB_2922892; West Grove, PA, USA), and incubated for 45 min. The membrane was analyzed in Li-Cor equipment (Li-Cor Biosciences; Lincoln, NE, USA) [68,69,70].

### 4.7. Collagen-Coated Polyacrylamide Substrate Preparation

Polyacrylamide gel substrates were prepared according to Wang and Pelham [71]. Briefly, No. 0 glass-bottom culture dishes (MatTek P35G-0-20-C; Ashland, MA, USA) were treated with 0.1 M NaOH, 97% (3-aminoproyl) trimethoxysilane (Sigma-Aldrich 281778; St. Louis, MO, USA), and 0.5% glutaraldehyde (Polysciences 01909; Warrington, PA, USA). Culture dishes were stored in a desiccator for up to 2 weeks until use. A 4.6 kPa hydrogel was made as follows: 100 µL of 40% acrylamide solution (Fisher Scientific BP1402; Waltham, MA, USA), 100 µL of 2% bis-acrylamide solution (Fisher Scientific BP1404; Waltham, MA, USA), 10 µL of 1 M 4-(2-hydroxyethyl) piperazine-1-ethanesulfonic acid (HEPES, Sigma-Aldrich H6147; St. Louis, MO, USA), 790 µL of deionized water, 6 µL of 1% ammonium persulfate (Bio-Rad 161-0700), and 4 µL of 0.4% (*v*/*v*) TEMED, (Fisher Scientific BP150; Waltham, MA, USA). Next, 4 µL of polymer solution was quickly pipetted onto the activated glass culture dishes and covered with a 12 mm No. 1.5 circular coverslip (Fisher Scientific 12-545-80; Waltham, MA, USA). Then, the coverslip was removed and washed three times with 50 nM HEPES. Finally, 1 mL of 200 mg/mL of type I collagen solution was added to the gel and the hydrogel was kept at 4 °C overnight.

### 4.8. Cell Migration Analysis

Individual cells were identified in each frame of the cell migration movies by using a previously described custom-written image segmentation algorithm in MATLAB (MathWorks, 2019a) [43].

### 4.9. Mouse Cerebellum Slices

Five- to seven-week-old C57BL/6J mice (The Jackson Laboratory, RRID:IMSR_JAX:000664) were terminally anesthetized in a CO_2_ chamber and then transcardially perfused with 1× DPBS (Gibco 14190-250; Thermo Fisher Scientific, Waltham, MA, USA). The cerebellum was extracted and transferred into chilled artificial cerebrospinal fluid (124 mM NaCl; Macron 7581-12, 2.5 mM KCl; Sigma-Aldrich P3911, 2.0 mM MgSO_4_; Sigma-Aldrich M3409, 1.25 mM KH_2_PO_4_; Sigma-Aldrich P5655, 26 mM NaHCO_3_; Sigma-Aldrich 93350, 10 mM glucose; Macron 4912-12). The cerebellum was sectioned into 300 µm coronal slices using a vibratome Ci 7000 smz (Campden Instruments; Lafayette, IN, USA). Cerebellum slices were submerged in Minimum Essential Media (MEM; Gibco 11095; Thermo Fisher Scientific, Waltham, MA, USA) supplemented with 15% fetal bovine serum (FBS; Gibco 26140079; Themo Fisher Scientific, Waltham, MA, USA), 100 mg/mL penicillin G, and 100 mg/mL streptomycin (Gibco 15140122; Thermo Fisher Scientific, Waltham, MA, USA) until use [72].

### 4.10. Cells Staining

Cells were stained with Vybrant cell-labeling solution (Invitrogen V22886; Thermo Fisher Scientific, Waltham, MA, USA) according to the manufacturer’s procedure. Briefly, cells were prepared at a density of 1 × 10^6^ cells/mL in media, and 5 µL of the cell-labeling solution was added per mL of cell suspension. Then, cells were incubated for 20 min. Cells were centrifuged at 1500 rpm for 5 min at room temperature, then resuspended in media and washed two more times [73].

### 4.11. Cerebellum-Slice Coculture with Medulloblastoma Cells

The cerebellum slice was transferred onto No. 0 glass-bottom 35 mm culture dishes (MatTek P06G-0-20-F; Ashland, MA, USA) containing MEM (MEM; Gibco 11095; Thermo Fisher Scientific, Waltham, MA, USA) supplemented with 15% FBS (FBS; Gibco 26140079; Thermo Fisher Scientific, Waltham, MA, USA), 100 mg/mL penicillin G, and 100 lg/mL streptomycin (Gibco 15140122; Thermo Fisher Scientific, Waltham, MA, USA). D425-OTX2OE, D425 control vector GFPOE, and D425-wt cells (500,000) were plated onto the cerebellum slice and cocultured for 24 h. The next day, 2.5 μL per 2 mL media of Iso-lectin GS-IB4 (Alexa Fluor 568; Molecular Probes I21412; Thermo Fisher Scientific, Waltham, MA, USA) [73] was added to the cerebellum slice to label the vasculature and incubated for 30 min. The cells were cocultured with the cerebellum slice for another 24 h.

### 4.12. Live-Cell Confocal Fluorescence Time-Lapse Imaging and Analysis of Cell Migration

The slice was imaged on a Zeiss LSM 7 Live swept-field laser confocal microscope (Carl Zeiss; Oberkochen, Baden-Württemberg, Germany) with a 20× 0.8 objective lens capable of simultaneous imaging in both green (GFP) (D425OTX2OE or D425 control vector GFPOE) and red (IB4 Alexa Fluor 568, Molecular Probes I21412; Thermo Fisher Scientific, Waltham, MA, USA). Maximal intensity projections from multiple z-stacks were used to generate 2D images for quantitative shape and motion analysis. The number of z-stacks was adjusted to ensure that the data acquisition of the whole slice was completed in under 10 min (eight planes with 10 mm of separation were used). The z-stacks were then imaged every 10 min for up to 24 h at 37 °C in a humidified 5% CO_2_ environment. Movies were recorded by time-lapse with intervals Δt of 10 min for 24 h. For each cell trajectory $\vec{r}$(t), the positions ${\vec{r}}_{j}=\vec{r}(t_{j})$, t_j_ = j∆t, j = 1, 2, 3, …, ∆t = 10 min were calculated, and the velocities $\left({\vec{V}}_{j})=({\vec{r}}_{j}-{\vec{r}}_{\left(j=1\right)}{)/(t}_{j}{-t}_{\left(j-1\right)}\right)$ were calculated.

### 4.13. siRNA Transfection

Cells were seeded onto 24-well plates (Sarstedt 83.3922; Numbrecht, Rhine-Westphalia, Germany) in triplicate per cell line and per condition. Cells, after 24 h, were transfected with 20 nM silencer siRNA oligonucleotides using lipofectamine RNAiMAX (Thermo Fisher Scientific 13778-075; Thermo Fisher Scientific, Waltham, MA, USA) according to the manufacturer’s procedure. The universal silencer negative control #2 siRNA (Ambion AM4613; Thermo Fisher Scientific, Waltham, MA, USA) was used as a negative control. Two different siRNAs were used to target the human OTX2 and mTOR siRNAs (Ambion OTX2, ID109382; Ambion mTOR ID145119; Thermo Fisher Scientific, Waltham, MA, USA), whose sequences are listed in Table S3. To assess knockdown efficiency, a separate set of transfections was performed and, seventy-two hours after transfection, total RNA was isolated from the transfected cells and RT-qPCR was performed to assess OTX2 or mTOR mRNA expression [69]. Three independent experiments were performed.

### 4.14. Gaussia Luciferase Activity

The day before transfection, 1.5 × 10^5^ cells in 500 μL growth medium were placed onto 24-well (Sarstedt 83.3922; Numbrecht, Rhine-Westphalia, Germany) plates in triplicate per cell line and per treatment. Cells were transfected with either mTOR promoter (GeneCopoeia HPRM45818-PG02; Rockville, MD, USA) or GAPDH (GeneCopoeia GAPDH-PG02; Rockville, MD, USA) reporter clones using Lipofectamine 3000 Transfection Reagent (Thermo Fisher Scientific L300-008; Thermo Fisher Scientific, Waltham, MA, USA) according to the manufacturer’s instructions. Briefly, 3.5 µg mTOR promoter and 2 µg GAPDH vector were mixed with 7 μL Lipofectamine 3000 and 5 μL P3000 in 0.3 mL OptiMEM reduced medium (Gibco 319855-070; Thermo Fisher Scientific, Waltham, MA, USA) and following 25 min incubation at room temperature, the mixture was added to the cells. After 48 h of transfection, 30 μL of the medium was taken in each assay, and luciferase activity was measured using the Pierce Gaussia Luciferase Glow Assay Kit (Thermo Fisher Scientific 16160; Thermo Fisher Scientific, Waltham, MA, USA) and a microplate reader/luminometer (Synergy, Biotek; Winooski, VT, USA) at 485 nm [52]. Two independent experiments were performed.

### 4.15. Cell Viability Assay

Cells were seeded onto 24-well plates (Sarstedt 83.3922; Numbrecht, Rhine-Westphalia, Germany) at a density of 5 × 10^4^ cells/well for 24 h in triplicate per cell line and per treatment. Then, the medium was replaced with fresh medium containing different concentrations of rapamycin inhibitors: PQR620 (MCE MedChemExpress HY-10026/CS-5948; Monmouth Junction, NJ, USA) or AZD8055 (Apexbio A8214; Houston, TX, USA). The working concentration of DMSO did not exceed 0.2%. After 48 h of treatment, cells were further stained with trypan blue (0.4%) (Gibco 15250-061; Thermo Fisher Scientific, Waltham, MA, USA) and then counted by using a Neubauer cell-counting chamber [68]. Three independent experiments were performed.

### 4.16. Bulk RNAseq

Total eukaryotic RNA isolates are quantified using a fluorometric RiboGreen assay. Total RNA integrity was assessed using capillary electrophoresis, generating an RNA Integrity Number (RIN). For samples to pass the initial QC step, they needed to quantify higher than 500 ng and have an RIN of 8 or greater. Total RNA samples were then converted to Illumina sequencing libraries. Library creation: Total RNA samples were converted to Illumina sequencing libraries using Illumina’s TruSeq RNA Sample Preparation Kit (RS-122-2001 or RS-122-2002) or Stranded mRNA Sample Preparation Kit (RS-122-2101). In summary, the mRNA from a normalized input mass of total RNA was isolated using oligo-dT-coated magnetic beads, fragmented, and then reverse-transcribed into cDNA. The cDNA was blunt-ended, A-tailed, and indexed by ligating molecularly barcoded adaptors. Libraries were amplified using 15 cycles of PCR. The final library size distribution was validated using capillary electrophoresis and quantified using fluorimetry (PicoGreen) and Q-PCR. Indexed libraries were then normalized, pooled, and size-selected to 320 bp (tight) using the PippinHT instrument. Cluster generation and sequencing: Pooled libraries were denatured and diluted to the appropriate clustering concentration. The libraries were then loaded onto the NovaSeq paired-end flow cell and clustering occurred on the instrument. Once clustering was complete, the sequencing reaction immediately began using Illumina’s 2-color SBS chemistry. Upon completion of read 1, a 7-base-pair index read was performed in the case of single-indexed libraries. If dual indexing was used during library preparation, 2 separate 8- or 10-base-pair index reads were performed. Finally, the clustered library fragments were re-synthesized in the reverse direction, thus producing the template for paired-end read 2. Then, 2 × 150 bp FastQ paired-end reads for 18 samples (n = 36.5 Million average per sample) were trimmed using Trimmomatic (v 0.33) enabled with the optional “-q” option: 3bp sliding-window trimming from the 3′ end requiring minimum Q30. Quality control on raw sequence data for each sample was performed with FastQC. Read mapping was performed via Hisat2 (v2.1.0) using the human genome (GRCh38) as a reference. Gene quantification was performed via Feature Counts for raw read counts. Differentially expressed genes were identified using the edgeR (negative binomial) feature in CLCGWB (Qiagen, Redwood City, CA, USA) using raw read counts. We filtered the generated list based on a minimum 2× absolute fold change and FDR-corrected *p* < 0.05.

### 4.17. Statistical Analysis

Data generated by incorporating specific statistical analysis for the correlative specimens are presented throughout the paper. Figure legends denote the number of samples from which data were obtained and the statistical method used. All data are represented as mean ± SD from at least two independent experiments. Three independent biological replicates per treatment and per cell line were performed per experiment. All analyses were performed using GraphPad Prism 9 Software.

## Data Availability

The data generated in this study are available upon request from the corresponding author.

## Supplementary Materials

The following supporting information can be downloaded at: https://www.mdpi.com/article/10.3390/ijms25084416/s1.
