# Supplementary material for: The OTX2 Gene Induces Tumor Growth and Triggers Leptomeningeal Metastasis by Regulating the mTORC2 Signaling Pathway in Group 3 Medulloblastomas
Source: Int J Mol Sci. 2024 Apr 17;25(8):4416. doi: 10.3390/ijms25084416 (PMC11050316; doi:10.3390/ijms25084416)
Supplement: Supplementary file 1 [file ijms-25-04416-s001.zip › Supplementary figures.pdf]

Supplementary Figures

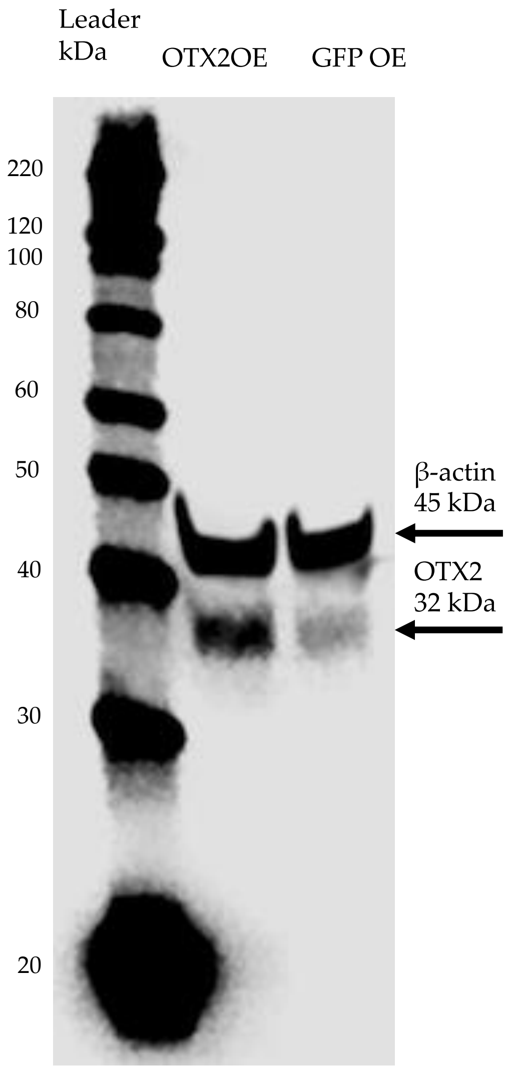

Supplementary Figure S1.
Western blot showing overexpression OTX2 in D425 cells.

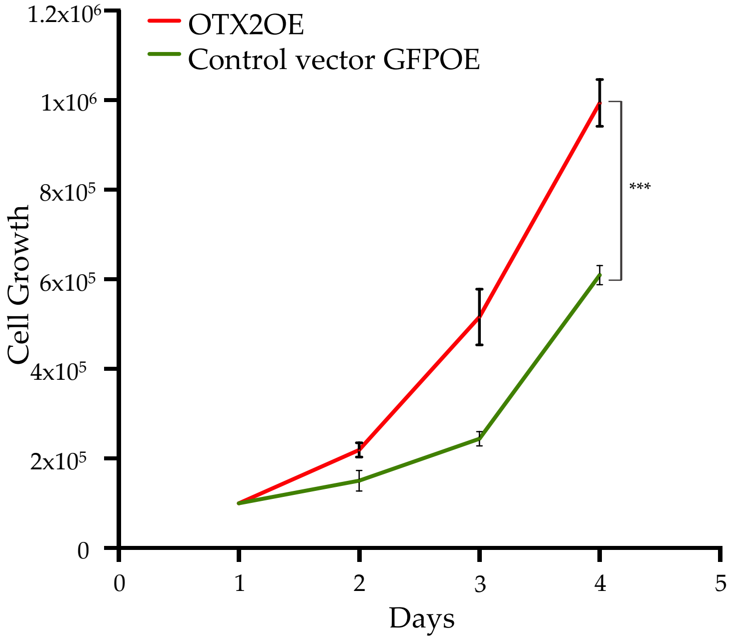

Supplementary Figure S2.
Overexpression of the OTX2 gene increases growth of D341 MB cells. $1X10^5$ D341 cells transduced either with OTX2OE or control vector GFPOE were plated and followed for cell growth at indicated days. Error bars show means ± SD and asterisks indicate significance compared to the control. ***P<0.0005 (Student's *t*-test).

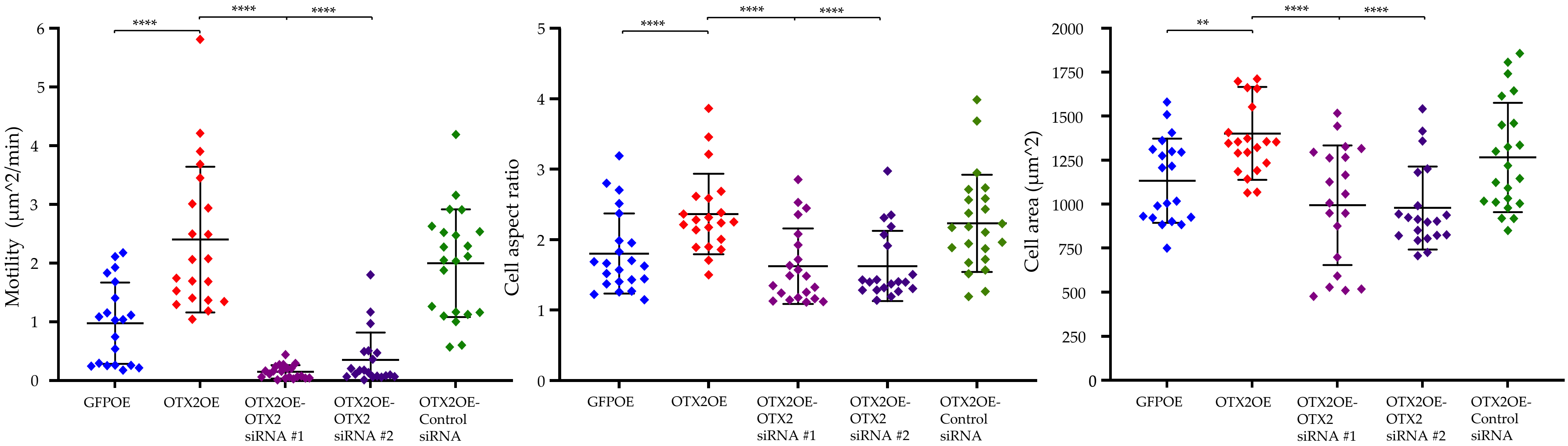

Supplementary Figure S3. Knockdown of OTX2 mRNA inhibits migration in D341 cells. Migration of D341 cells transduced either with the OTX2 overexpression vector (OTX2OE) or GFPOE and transfected with OTX2 or control siRNAs. After 48 hours of transfection cells were placed on 4.6 kPa PAGs coated with type I collagen. Cells were monitored in a Nikon Ti2 microscope and were recorded time-lapse with intervals of 10 min for 16 h. The random motility coefficient **(A)**, cell aspect ratio (**B**), and (**C**) cell area of about twenty and twenty-five individual cells per treatment were calculated using a customized MatLab script. Two independent siRNAs directed against the OTX2 mRNA were used. Error bars indicate means ± SEM, and asterisks indicate significance compared to the control plasmid. **P<0.005, ***P<0.0, ****P < 0.00005. (Student's *t-test*).

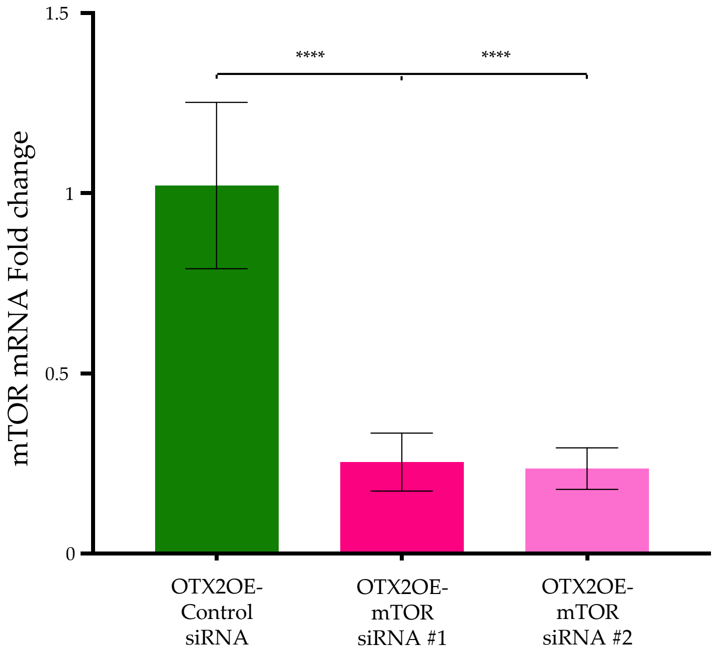

Supplementary Figure S4: Gene silencing of the mTOR mRNA using siRNA technology. qPCR was used to confirm the transient knockdown of the mTOR mRNA using small siRNAs. The obtained values were normalized to the housekeeping gene hb-actin. Statistical significance was determined using Student's *t-test* (***P<0.00005). Error bars indicate means ± SD. Two independent experiments were carried out, and three biological replicates were performed per each.

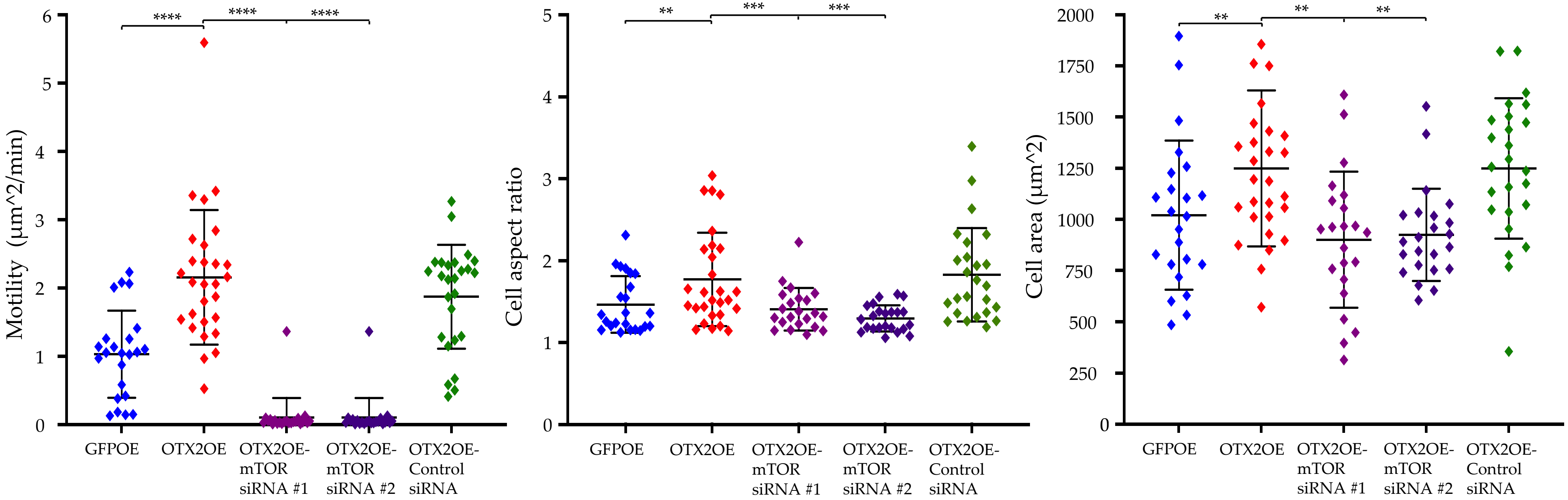

Supplementary Figure S5. Knockdown of mTOR mRNA inhibits migration in D341 cells. Migration of D341 cells transduced with the OTX2 overexpression plasmid (OTX2OE) or GFPOE as a control and transfected with OTX2 siRNA. After 48 hours of transfection cells were placed on 4.6 kPa PAGs coated with type I collagen. Cells were monitored in a Nikon Ti2 microscope and were recorded time-lapse with intervals of 10 min for 16 h. The random motility coefficient (**A**), cell aspect ratio **(B)**, and (**C**) cell area of twenty and twenty-five individual cells per treatment were calculated using a customized MatLab script. Two independent siRNAs directed against the mTOR mRNA were used. Error bars indicate means ± SEM, and asterisks indicate significance compared to the control plasmid. **$P<0.005$, ***$P<0.0005$, ****$P < 0.00005$. (Student's *t-test*).
