# Supplementary material for: The OTX2 Gene Induces Tumor Growth and Triggers Leptomeningeal Metastasis by Regulating the mTORC2 Signaling Pathway in Group 3 Medulloblastomas
Source: Int J Mol Sci. 2024 Apr 17;25(8):4416. doi: 10.3390/ijms25084416 (PMC11050316; doi:10.3390/ijms25084416)
Supplement: Supplementary file 1 [file ijms-25-04416-s001.zip › Supplementary Tables S1-S3.pdf]

Table S1

	mTOR migratory genes	
Gene symbol	Protein name	Fold change
RHOH	Ras homolog family member H	7.90302208
TCTE1	t-complex-associated-testis-expressed 1	5.03275079
SPATA4	Spermatogenesis associated 4	3.52173763
FLT1	Fms related receptor tyrosine kinase 1	3.21995989
MYO5C	Myosin VC	3.01310208
RSPH3	Radial spoke head 3	2.91353403
CAPG	Capping actin protein, gelsolin like	2.89424259
MEIG1	Meiosis/spermiogenesis associated 1	2.67957714
MID1	Midline 1	2.61454996
FNBP1L	Formin binding protein 1 like	2.57540571
ADAM8	ADAM metallopeptidase domain 8	2.55443531
STOX1	Storkhead box 1	2.48273568
CLIP3	CAP-Gly domain containing linker protein 3	2.46877779
CDC42EP3	CDC42 effector protein 3	2.3729356
SPIN1	Spindlin 1	2.28779334
EVI5	Ecotropic viral integration site 5	2.27902084
WDR66	Cilia and flagella associated protein 251	2.22487289
TRIP11	Thyroid hormone receptor interactor 11	2.21789854
LATS2	Large tumor suppressor kinase 2	2.21493333
BMF	Bcl2 modifying factor	2.21178766
SLAIN1	SLAIN motif family member 1	2.20076231
CHMP4A	Charged multivesicular body protein 4A	2.19732174
RMDN2	Regulator of microtubule dynamics 2	2.19429655
RSPH4A	Radial spoke head component 4A	2.1941541
CCNB3	Cyclin B3	2.17413257
PFN4	Profilin family member 4	2.15908668
FILIP1	Filamin A interacting protein 1	2.14084054
CLIP1	CAP-Gly domain containing linker protein 1	2.13441998
PGM5	Phosphoglucomutase 5	2.12866312
IFT43	Intraflagellar transport 43	2.11524978
B9D2	B9 domain containing 2	2.11041237
CALB1	Calbindin 1	2.10572869
KIFAP3	Kinesin associated protein 3	2.08948629
RELB	Proto-oncogene, NF-kB subunit	2.06572781

TFDP2	Transcription factor Dp-2	2.065508
PDE4DIP	Phosphodiesterase 4D	2.05145025
FERMT2	FERM domain containing kindlin 2	2.01945389
ENKUR	Enkurin, TRPC channel interacting protein	2.01500987
EFHB	EF-hand domain family member B	2.00936588
BMERB1	bMERB domain containing 1	2.00881986
TNNI1	Troponin I1, slow skeletal type	2.00711613
CGN	Cingulin	2.00588654
TUBA3C	Tubulin alpha 3c	-3.3390037
ABCA2	ATP binding cassette subfamily A member 2	-3.1385977
SEPTIN1/44805	Septin 1	-3.0849846
SHROOM2	Shroom family member 2	-2.8106402
PPL	Periplakin	-2.6933733
ACTN2	Actinin alpha 2	-2.6319658
DRD4	Dopamine receptor D4	-2.5231404
SLC4A1	Solute carrier family 4 member 1	-2.4951579
XIRP2	Xin actin binding repeat containing 2	-2.4635309
LRRC45	Leucine rich repeat containing 45	-2.4302219
FSD1	Fibronectin type III and SPRY domain containing 1	-2.4090195
TNNC2	Troponin C2, fast skeletal type	-2.3479093
ACTA1	Actin alpha 1, skeletal muscle	-2.3340937
MARVELD1	MARVEL domain containing 1	-2.2974567
RILP	Rab interacting lysosomal protein	-2.289113
DGKQ	Diacylglycerol kinase theta	-2.2825598
NPM3	Nucleophosmin/nucleoplasmin 3	-2.2657574
SYCE1L	Synaptonemal complex central element protein 1 like	-2.2118757
CCDC124	Coiled-coil domain containing 124	-2.2075697
NUDT21	Nudix hydrolase 21	-2.1761027
TEC	Tec protein tyrosine kinase	-2.1715085
HASPIN	Histone H3 associated protein kinase	-2.1585964
SPATC1L	Spermatogenesis and centriole associated 1 like	-2.1540213
SYNPO	Synaptopodin	-2.143688
ACADS	acyl-CoA dehydrogenase short chain	-2.1406835
DIXDC1	DIX domain containing 1	-2.1274197
HIP1R	Huntingtin interacting protein 1 related	-2.0924438
MYH10	Myosin heavy chain 10	-2.090922
HLA-DRB1	Major histocompatibility complex, class II, DR beta 1	-2.0796544
LMNB2	Lamin B2	-2.0767632
POC5	POC5 centriolar protein	-2.0651481

BCL2L11	BCL2 like 11	-2.0580241
KIFC2	Kinesin family member C2	-2.0440324
WHRN	Whirlin	-2.023871
MSRA	Methionine sulfoxide reductase A	-2.0149004
ACTR3B	Actin related protein 3B	-2.011168
PRKCE	Protein kinase C epsilon	-2.003062

Table S2

**Name**	**Forward 5'-3'**	**Reverse 5'-3'**
Target of rapamycin kinase (MTOR)	CCTTAATTTGTTGCCCCGCT	CCTCGCGTGCTGGACATC
DEP domain containing MTOR interacting protein (DEPTOR)	CGGCTGATGAGCCCTGA	ATGTGTCCAACAAGCACCC
Regulatory associated protein of MTOR complex 1 (PROTOR)	CGGCTGAGAGCATTGGACT	GTGGACAGCGAGCTGGTG
AKT1 substrate 1 (AKT1S1) (PRAS40)	CCACAGAGACAGAGACCTCC	GAGTACAGATGATGGCAGCCT
MTOR associated protein, LST8 homolog (mLST8)	GGATCTTCCAGGTGAACGCA	CTCATCGTGGGTGACCAGAG
RPTOR independent companion of MTOR complex 2 (RICTOR)	TGTCACAACTGGGATGCTGT	TTGGAGGATGACCGGTTTGG
Proline rich 5 like (PRR5L) (Protor 2)	TCCCTACCCTGCAGGCAATA	CAGAGTGTTCACGAGCCCAC
MAPK associated protein 1 (MAPKAP1) (mSin1)	TCTACCTCCCTCTGCACTCG	GGCTCATCTGCTGGCAGTAT
AKT serine/threonine kinase 1 (AKT1)	GCGTGACCATGAACGAGTTT	CACACACTCACCGAGAACCG
AKT serine/threonine kinase 2 (AKT2)	GCAGAGATTGTCTCGGCTCT	AAAACCTTCTGTGGGACCCC
AKT serine/threonine kinase 3 (AKT3)	GGATGCCTCTACAACCCATCA	AGGATGAAGTGGCACACACT
ras homolog family member H (RHOH)	ACAAGCCCACAGTGTACGAG	GCTACTCTGTGGCCAACCAT
Myosin VC (MYO5C)	CGCAGCTCTCTGTACTTGCT	GCACAGAGCTACCCTTCCAG
CDC42 effector protein 3 (CDC42EP3)	GCCAGTTCCCTGGGCATAAT	ACCATTGGAGGATCCCAAGC

Table S3

Name	Sequence 5'->3'	
	Sense	Antisense
OTX1 si RNA #1	GGAUAUGCUGGCUCAACUUtt	AAGUUGAGCCAGCAUAUtt
OTX1 si RNA #2	GGCUUCAGGUUAUAGUCAAtt	UUGACUAUAACCUGAAGCCtg
mTOR siRNA #1	CCCAGGUGUGAUCAAUAAUtt	AUUAUUGAUCACACCUGGGtt
mTOR siRNA #2	GGGCAUGAAUCGGGAUGAUtt	AUCAUCCCGAUUCAUGCCCtt
